# Rehabilitation Strategies for a Patient With Traumatic Multiple Fractures: A Case Report

**DOI:** 10.7759/cureus.29732

**Published:** 2022-09-29

**Authors:** Kamya J Somaiya, Shubhangi Patil, Rupali Thorat

**Affiliations:** 1 Department of Physiotherapy, Ravi Nair Physiotherapy College, Datta Meghe Institute of Medical Sciences, Wardha, IND

**Keywords:** lower limb weakness, edema, pain, restricted mouth opening, trismus, case report, quality of life, physiotherapy rehabilitation, thoracolumbar fracture, le fort fracture

## Abstract

Le Fort fractures are a specific kind of facial bone fracture that develops after a blow to the face. Most of the fractures of the spine occur in the thoracolumbar region. The benefits of physiotherapy, which includes manual therapies and exercise regimens, for patients are becoming more and more clear. We are going to report the case of a 25-year-old male adult with a thoracolumbar fracture and a Le Fort fracture. We made an effort to develop a post-surgical physical therapy rehabilitation program. The patient's condition and general quality of life were successfully improved. We focused on the patient's primary symptoms, which were thoracolumbar discomfort, lower limb weakness, edema and pain on the left side of the face, trismus, and restricted mouth opening. We worked on the complaints mentioned by the patient and were successful in resolving them.

## Introduction

Le Fort fractures are a type of facial bone fracture that occurs as a result of blunt facial trauma (most usually from a car accident, an assault, or a fall) [1]. It is a subset of injuries that cause discontinuity of the midface, which is made up of the maxilla, inferolateral orbital rims, sphenoids, ethmoids, and zygomas, as well as rupture of the facial buttresses, which provide the facial skeleton strength and rigidity. Le Fort types I, II, and III classification depends on the involvement of the maxillary, nasal, and zygomatic bones [1]. Le Fort I consists of fractures of the maxilla immediately above the upper dental arch. Le Fort II consists of fractures of the frontal-nasal and maxillary structures. Le Fort III consists of combined fractures of the frontal, middle, and posterior maxillary and nasal structures [1]. Fractures of the thoracolumbar spine continue to be a significant source of potential morbidity. Advances in treatment have reduced the invasiveness of our procedure, and in some stable conditions, it has been completely eliminated [2]. 

Le Fort fractures accounted for around six percent of all facial fractures. The thoracolumbar area is responsible for 90% of all spine fractures. The majority of thoracolumbar injuries happen between the 11th thoracic vertebra (T11) and the second lumbar vertebra (L2), which is the biomechanically weakest area for stress [3]. There is growing evidence that physiotherapy, which includes manual treatments and exercise programs, can help patients [4].

We are presenting a case of a 25-year-old male adult with Le Fort fracture and thoracolumbar fracture. We attempted to create a post-operative physiotherapy rehabilitation program that was successful in improving the patient's condition and overall quality of life. We concentrated on the patient's main symptoms, which were pain and edema on the left side of the face, trismus and restricted mouth opening, thoracolumbar pain, and lower limb weakness. To assess the efficiency of the treatment program, we used a variety of outcome measures such as the Lower Extremity Functional Scale for lower extremity function, Inter-Incisional Opening Distance - Metal Ruler technique for mouth opening, Manual Muscle Testing, and Numerical Pain Rating Scale.

## Case presentation

Patient information

In this case, the patient is a 25-year-old male student with a dominant right hand. The patient met with a road traffic accident (RTA) on October 23. The patient was first sent to a private hospital, where he received primary treatment. The patient was then referred to Government Medical College and Hospital (GMC), Nagpur, India, where the contused lacerated wound was sutured. On October 23, the patient was transferred to Acharya Vinoba Bhave Rural Hospital (AVBRH), Wardha, India, Casualty for further treatment, which included a CT Head with 3D Reconstruction and an MRI of the lumbar spine on October 25 and 26, respectively. A CT head scan indicated a comminuted displaced fracture of the bilateral maxillary sinus, as well as a left orbit fracture, while an MRI of the lumbar spine revealed a compression fracture at the L1 vertebra level, as well as left paracentral disc herniation at the L1-L2 level. The final diagnosis was High Le Fort I and left infraorbital rim fracture, as well as compression fracture at the L1 vertebra and left paracentral disc herniation at the L1-L2 level, based on the studies. On November 22, open reduction and internal fixation of bilateral Le-Fort I fractures utilizing titanium plates and intermaxillary fixation (IMF) screws were used to treat the high Le Fort I and left infraorbital rim fractures, while the L1 vertebral fracture was treated conservatively with a Taylor brace. Pain history revealed a traumatic cause of pain, which was sudden in onset, dull aching in nature, aggravated by manipulation, and self-relieved. The main complaints of the patient were pain and edema on the left side of the face, trismus and restricted mouth opening, thoracolumbar pain, and lower limb weakness.

Clinical findings

The physical examination was conducted after the patient gave his consent. During the physical evaluation, the patient appeared to be alert, attentive, and well-oriented in terms of time, location, and person. During the assessment, the patient was in a sitting position. The heart rate was 70 beats per minute, and the respiratory rate was 18 breaths per minute. There was no pallor, icterus, clubbing, cyanosis, or edema feet on evaluation. There was no deformity, no loss of muscle mass, and no scar on assessments. On palpation, back muscles including paraspinal muscles, and neck muscles including deep cervical muscles were in spasm, and the left side of the face was swollen due to trauma. Examination of the musculoskeletal system revealed a reduction in the strength of all the major lower limb muscles. On manual muscle testing, the left lower limb graded two and the right lower limb graded three out of five. Back muscles, i.e., paraspinal muscles and neck muscles were in spasm as well as tenderness was found at the L1 level. Back pain and neck pain are measured on the Numerical Pain Rating Scale (NPRS) scale. The rating for back pain was seven out of 10 and for neck pain six out of 10. Mouth opening was also affected due to trauma. On maxillofacial examination, step and tenderness were found present over the left infraorbital rim and tenderness over the left malar region and bilateral zygomaticomaxillary buttress region. On intraoral examination, mouth opening was 20 mm approx. The other system examination which included respiratory and cardiovascular systems was also carried out, but no significant findings were found.

Therapeutic intervention

**Figure 1:**
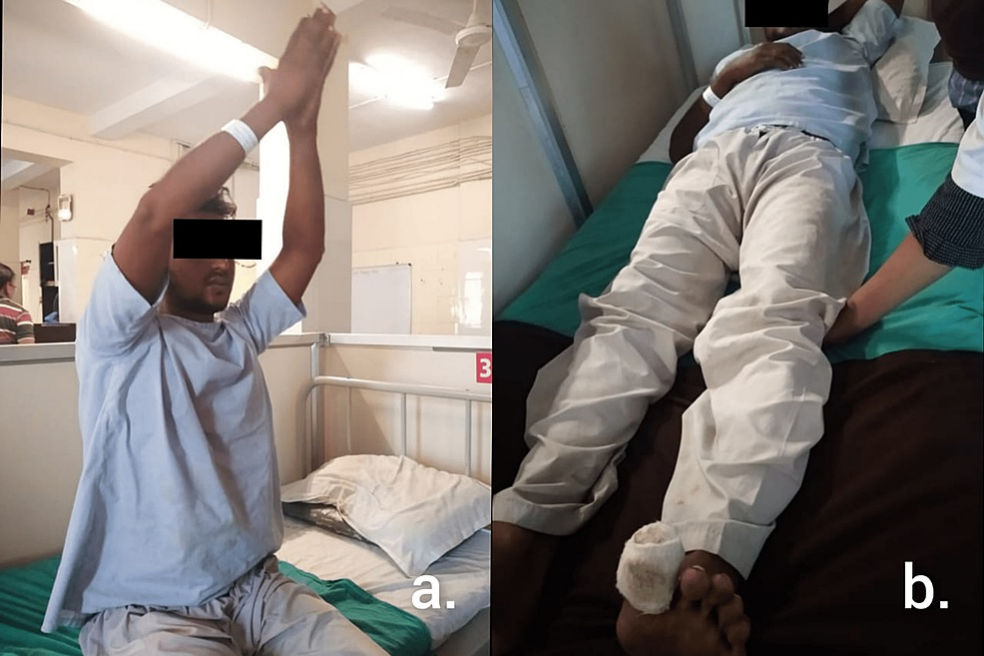
Shows the exercises performed by the patient In the figure, a. shows the thoracic expansion exercise done by the patient, and b. shows static quads done by the patient.

The therapeutic intervention was given for four weeks. Table 1 explains the treatment protocol for week one and week two. Table 2 explains the treatment protocol for week three and week four. Figure 1 shows the exercises performed by the patient. 

**Table 1:** Treatment protocol followed for week one and week two TJE=therapeutic jaw exercises

Treatment protocol for week one and week two		
Goals	Therapeutic intervention	Regimen
Patient education	Counseling the patient about his condition and the need for physiotherapy treatment.	Week one – At the beginning of the session, the management was explained to the patient.
To reduce pain and edema over the left side of the face	Cryotherapy	Week one – Cold pack application for 10 minutes. It was applied three times a day.
To reduce trismus and to increase the mouth opening	Oral motor exercises for the jaw	Week one – Oral exercises and TJE – One set of 10 reps given.
	Muscle energy technique (MET)	MET – One set of four reps was employed.
	TJE	Week 2 – Oral exercises and TJE – Two sets of 10 reps given.
	According to the patient’s condition, the techniques are given.	MET – Two sets of four reps were employed.
For avoiding chest complications and to keep the chest clear	Diaphragmatic breathing exercise (DBE)	Week one – DBE – One set of 10 reps was applied for exercise.
	Thoracic expansion exercise (TEE) – Shown in Figure 1a	Week two – TEE – One set of 10 reps was applied for exercise.
Postural education for the thoracolumbar fracture	Postural education is employed to the patient by the therapist.	Week one and week two – The following precautions were followed: emphasizing neutral spine, avoidance of forward stooped posture, and no motion given to spine.
To increase the strength of the lower limb muscles and abdominals	Abdominal isometrics – Static abdominals	Week one – One set of 10 reps of each exercise was employed.
	For quads, hams, calf – Static quads – Shown in Figure 1b	
	Static hams	Week two – Two sets of 10 reps of each exercise were employed.
	Ankle Isotonics – Ankle pumps	
To initiate weight bearing	Ambulation with the help of a walker was initiated.	Week one and week two – The patient was taught to walk with the walker according to his ability. The patient was encouraged to sit. Sitting and walking were carried out for 10 minutes and three times a day and the time was increased according to the tolerance of the patient.

**Table 2:** Treatment protocol followed for week three and week four

Treatment Protocol for week three and week four. The same protocol as for weeks one and two and a few additional interventions		
Goals	Therapeutic intervention	Regimen
To strengthen the facial skeleton and the muscles	Oral motor exercises for the jaw with resistance were employed.	Week three – One set of 10 reps of each exercise was performed.
		Week four – Two sets of 10 reps of each exercise were performed
To relieve the tightness of the neck muscles, especially the upper trapezius	Myofascial release (MFR) for upper trapezius muscle	Three sessions were given on alternate days. After each session cryotherapy was employed.
To maintain strength and increase the endurance of the lower limb muscles and abdominals	Abdominal isometrics – Static Abdominals	Week three – One set of 10 reps of each exercise was performed.
	For quads, hams, and calf – Multiple angle isometrics for quads	
	Multiple angle isometrics for hams	Week four – Two sets of 10 reps of each exercise were performed.
	Ankle isotonic – Ankle pumps	
To increase the mobility of the thoracolumbar spine	Active range of motion exercises including active extension is employed. Bridging was initiated.	Week three – One set of 10 reps was employed for each exercise
		Week four – Two sets of 10 reps were employed for each exercise
To continue weight bearing	Walking with the help of a cane was employed.	Week three – Ambulation for 10 min according to the tolerance of pain was employed.
		Week four – Independent ambulation for 10 min according to the tolerance of the patient was employed.

Follow-up and outcomes

**Figure 2:**
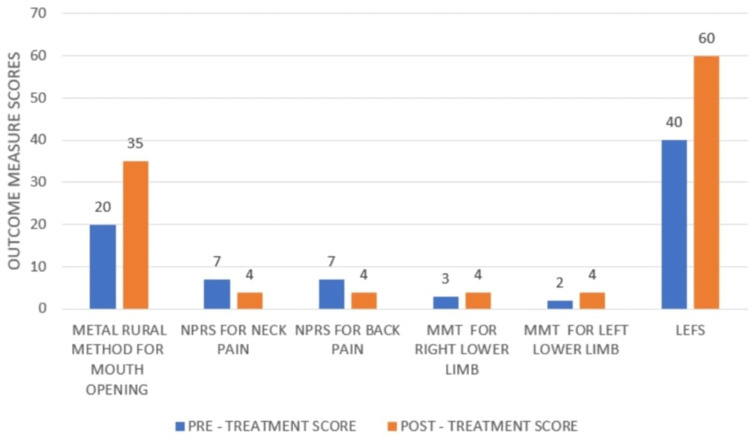
The pre-treatment and post-treatment outcome measure scores Inter-Incisional Mouth Opening Distance - Metal Rural Method We used a direct method of measuring the inter-incisional distance. The patient was asked to open their mouth as wide as possible. The measurement was acquired three times by placing a plastic ruler between the mandibular and maxillary central incisors, and a mean of the three was taken as the value. NPRS=Numerical Pain Rating Scale The Numerical Pain Rating Scale (NPRS) (an outcome measure) is a unidimensional measure of pain intensity in adults, including those with chronic pain. It is a ten-point scale where 0 represents 'no pain' and 10 represents 'worse pain.' MMT=Manual Muscle Testing It is a widely accepted method for evaluating muscle strength. We utilized the Oxford Scale for measuring the strength of the muscles. LEFS=Lower Extremity Functional Scale It is a patient-related outcome measure (PROM) that is utilized for measuring the lower extremity function. It is a questionnaire containing 20 questions related to the patient's daily activities.

The patient came for a follow-up after four weeks of the therapy session. When the patient came for follow-up, the assessment was done after the patient’s consent. Figure 2 displays the outcome measures scores of the patient. The results were evaluated based on the outcome measure scores. 

## Discussion

Wang et al. conducted research that supports the intervention program's effectiveness in reducing trismus and mandibular function deficits in individuals undergoing curative surgery for oral cancer. This study included the following interventions: jaw exercises, masticatory muscle massage, and warm compress [5]. A study conducted by Rasostra et al. showed the efficacy of therapeutic intervention in treating trismus [6]. A study conducted by Senthilkumar et al. found that physiotherapy therapies were beneficial in reducing trismus [7]. In chronic temporomandibular joint dysfunction (TMJD) patients, both muscle energy technique (MET) and myofascial release (MFR) are beneficial in lowering discomfort and increasing range of motion (ROM); however, MET was found to be superior to MFR [8]. 

Patients with neck discomfort benefitted from a well-organized and targeted physical therapy program [9]. In a study on neck pain patients, Moffett et al. demonstrated the value of a good physiotherapy regimen in reducing such patients' discomfort [10]. MFR has been associated with significant improvements in pain, flexibility, ROM, and quality of life (QOL) [11]. In a study, Groenewag et al. highlighted the value of manual therapy and physical therapy for people with chronic neck discomfort [12]. Mckinney et al. conducted a study in which they concluded the effectiveness of physiotherapy in patients with acute neck pain following trauma. [13] 

Lumbar discomfort is three times more likely in persons with diminished muscular strength. As a result, exercises for improving the strength of the back muscles are one of the most common therapy options for back pain. These exercises include static back, static adductors, and pelvic bridging. They seek to enhance posture, trunk muscular strength, and aerobic capacity, resulting in less discomfort and improved functional status [14]. Patients with compression fractures of the thoracic and lumbar spine should receive brace treatment along with further physical therapy [15]. Sridharan et al. conducted a case study to determine the importance of physiotherapy management in patients with maxillofacial trauma. [16]. According to research by Mackay et al., people with brain injuries who receive effective rehabilitation programs have higher QOL and are able to resume their activities of daily living (ADLs) more quickly [17].

In this way, many studies conclude the effectiveness of various physiotherapy interventions in improving the quality of life of patients with various medical conditions. This case study was conducted to assess the efficacy of physiotherapy intervention in a patient who had a Le Fort fracture I as well as a thoracolumbar fracture at the L1 level, and it provides evidence of the efficacy of a physiotherapy treatment regimen in such patients. The therapy procedure lasted for four weeks and was quite effective in improving the patient's health and even his quality of life.

## Conclusions

The patient's therapeutic design was found to be very successful. The patient's principal complaints included trismus and limited mouth opening, pain in the thoracolumbar area, weakness in the lower limbs, and pain and edema on the left side of the face. We concentrated on these concerns. For each concern, we used a combination of physiotherapy interventions to improve and minimize it. We worked on these concerns and discovered that they were successfully reduced, leading to an improvement in the patient's quality of life. The pain in the back, the strength of both lower limbs, the mouth opening, the pain and edema on the left side of the face as well as the trismus were all much improved by our treatment. Though few studies proved the effectiveness of physiotherapy regimen in patients with maxillofacial trauma, we presented a four-week designed protocol that was beneficial in such patients and could be applied further. According to the findings of our study, the patient with a thoracolumbar fracture and maxillofacial trauma responded well to our physiotherapy treatment plan.
